# The Impact of Human DNA Glycosylases on the Activity of DNA Polymerase β toward Various Base Excision Repair Intermediates

**DOI:** 10.3390/ijms24119594

**Published:** 2023-05-31

**Authors:** Artemiy S. Bakman, Stanislav S. Boichenko, Aleksandra A. Kuznetsova, Alexander A. Ishchenko, Murat Saparbaev, Nikita A. Kuznetsov

**Affiliations:** 1 Institute of Chemical Biology and Fundamental Medicine, Siberian Branch of Russian Academy of Sciences (SB RAS), 8 Prospekt Akad. Lavrentyeva, Novosibirsk 630090, Russia; 2 Department of Natural Sciences, Novosibirsk State University, 2 Pirogova Str., Novosibirsk 630090, Russia; 3 Group «Mechanisms of DNA Repair and Carcinogenesis», Gustave Roussy Cancer Campus, CNRS UMR9019, Université Paris-Saclay, 94805 Villejuif, France

**Keywords:** DNA repair, apurinic/apyrimidinic endonuclease, DNA–protein interaction, protein–protein interaction, damaged DNA transfer, conformational change, fluorescence, pre-steady-state kinetics

## Abstract

Base excision repair (BER) is one of the important systems for the maintenance of genome stability via repair of DNA lesions. BER is a multistep process involving a number of enzymes, including damage-specific DNA glycosylases, apurinic/apyrimidinic (AP) endonuclease 1, DNA polymerase β, and DNA ligase. Coordination of BER is implemented by multiple protein–protein interactions between BER participants. Nonetheless, mechanisms of these interactions and their roles in the BER coordination are poorly understood. Here, we report a study on Polβ’s nucleotidyl transferase activity toward different DNA substrates (that mimic DNA intermediates arising during BER) in the presence of various DNA glycosylases (AAG, OGG1, NTHL1, MBD4, UNG, or SMUG1) using rapid-quench-flow and stopped-flow fluorescence approaches. It was shown that Polβ efficiently adds a single nucleotide into different types of single-strand breaks either with or without a 5′-dRP–mimicking group. The obtained data indicate that DNA glycosylases AAG, OGG1, NTHL1, MBD4, UNG, and SMUG1, but not NEIL1, enhance Polβ’s activity toward the model DNA intermediates.

## 1. Introduction

Cellular genomic DNA continuously undergoes damage due to endogenous and exogenous factors [1]. An important system maintaining genome stability in mammalian cells is base excision repair (BER), which prevents premature aging, cancer, and many other human health problems by repairing DNA lesions [2,3,4]. DNA lesions as base modifications, base losses, and single-strand breaks (SSBs) can be processed by BER [5]. BER in mammalian cells is a multistep process that involves a number of enzymes, including damage-specific DNA glycosylases, apurinic/apyrimidinic (AP) endonuclease 1, DNA polymerase β, and DNA ligase.

Typically, BER is initiated by a spontaneous base loss or by DNA glycosylase’s cleaving the *N*-glycosidic bond of a damaged base, thus forming an apurinic/apyrimidinic (AP) site [5,6]. Subsequently, APE1 incises the damaged strand on the 5′ side of the abasic site, thereby generating a 1 nt gap with 3′-hydroxyl and 5′-deoxyribose phosphate (dRP) groups at the ends [7]. Then, Polβ fills the gap using the undamaged strand as a template and removes the 5′-dRP group by its intrinsic 5′-dRP-lyase activity [8,9,10]. To complete DNA repair, the SSB is sealed by DNA ligase [5]. If the 5′ end is blocked and cannot be processed by the lyase activity of Polβ, then BER can be executed as its long-patch subpathway [11,12,13]. In this case, Polβ also adds a single nucleotide to the 3′ end of the DNA, after which Polδ continues strand displacement synthesis in the presence of proliferating cell nuclear antigen (PCNA) and replication factor C (RFC) [5]. The resulting flap of 2–12 nucleotides is cut off by flap endonuclease 1, and the final nick is sealed by DNA ligase I [5,14].

It is believed that BER coordination is implemented by multiple protein–protein interactions involving both DNA repair enzymes and accessory proteins such as X-ray repair cross-complimenting protein 1 (XRCC1) and PCNA [5]. The protein–protein interactions in BER have been intensively studied in the last two decades as reviewed elsewhere [5,15,16,17]. There are two major mechanisms underlying the coordination of BER, as discussed in the literature [5]. One of these mechanisms is the “passing the baton” model, according to which, DNA intermediates of the BER pathway are passed on from one protein to the next in a coordinated manner. Judging by this model, BER enzymes can form transient protein–protein contacts on the damaged DNA. Another mechanism implies the existence of preassembled complexes between DNA repair proteins. Many experimental findings support both the “passing the baton” model and the model of preassembled stable multiprotein repair complexes and suggest that these mechanisms occur in live cells and participate in the coordination of BER [15].

Thus, knowledge about protein–protein interactions between BER participants is required for a deeper understanding of this pathway. In spite of numerous recent studies on this topic, many aspects of the BER coordination remain unknown or poorly understood. One of such aspects is the problem of interactions between Polβ and DNA glycosylases. In humans, there are 11 known DNA glycosylases, which can be classified by their substrate specificity, structural organization, and the presence of AP-lyase activity (Table 1). It has been shown that some DNA glycosylases with different types of structural organization and different damage specificity can specifically interact with Polβ (Table 1). Nonetheless, mechanisms of these interactions and their roles in the BER coordination are poorly investigated.

To clarify how interactions between Polβ and DNA glycosylases may participate in BER coordination, we decided to study the effects of various DNA glycosylases on Polβ’s nucleotidyl transferase activity toward different DNA substrates corresponding to different BER intermediates. For this purpose, we chose seven human DNA glycosylases: uracil-N glycosylase (UNG), single-strand-specific monofunctional uracil DNA glycosylase 1 (SMUG1), methyl-binding domain glycosylase 4 (MBD4), 8-oxoG DNA glycosylase 1 (OGG1), endonuclease III-like 1 (NTHL1), methylpurine glycosylase (AAG, also referred to as MPG), and endonuclease VIII-like glycosylase 1 (NEIL1). As readers can see in Table 1, some of these DNA glycosylases have already been reported to interact with Polβ, while others have not. These seven DNA glycosylases cover all known structural superfamilies of human DNA glycosylases and their broad spectrum of specificity to lesions. The experimental results obtained in this study provide new information about effects of different DNA glycosylases on Polβ-catalyzed nucleotidyl transferase activity. We showed that DNA glycosylases of different substrate specificity and structural organization can enhance Polβ’s activity toward different BER intermediates. These results advance our understanding of the coordination of BER and suggest that DNA glycosylases may participate not only in the early stages of this pathway, but also in the gap-filling reaction to form contacts with Polβ. We discuss our results in the context of existing models of BER coordination.

## 2. Results and Discussion

### 2.1. Interaction of Polβ with Model DNA Substrates

**Figure 1:**
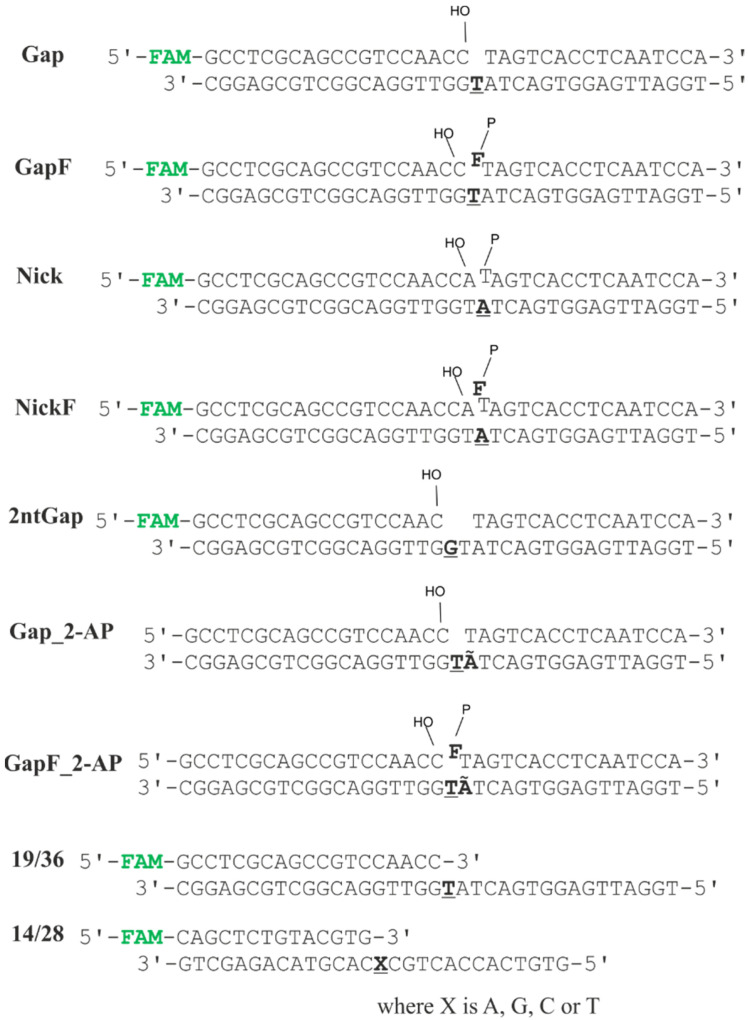
Structures of the DNA substrates used in this work. **F** is a 3-hydroxy-2-hydroxymethyltetrahydrofuran residue, **Ã** is 2-aminopurine, and **FAM** is a 6-carboxyfluorescein residue. The templating nucleotide is boldfaced and underlined. At the ends of SSBs, a 3′-hydroxyl group is depicted as “HO–” and a 5′-phosphate group is displayed as “P–”.

We tested Polβ polymerase activity on model DNA substrates (Figure 1) that imitate DNA intermediates arising during the BER process in live cells. GapF is a model of a BER intermediate deriving from AP site-containing DNA after its processing by APE1. Therefore, it is a canonical substrate of Polβ during BER. NickF is formed from GapF when Polβ incorporates the first nucleotide into the latter. Despite the distributive mode of action of Polβ, it can be hypothesized that Polβ can incorporate a second nucleotide into the SSB generated by APE1, thereby leading to the long-patch subpathway of BER. It is worth noting that a 5′-F group (a tetrahydrofuran residue imitating a 5′-dRP residue) cannot be processed by the 5′-deoxyribose phosphate lyase activity of Polβ. Gap and Nick are models of two types of SSBs: gapped and nicked DNA, respectively. These types of SSB can also emerge during BER. For instance, gapped DNA can form after the action of bifunctional glycosylase (Neil, for example) on DNA containing an AP site and subsequent cleaning up of a 3′-phosphate by APE1 owing to its 3′-phosphatase activity. When Polβ fills this gap by incorporation of one nucleotide, nicked DNA is generated. Besides, gapped and nicked DNA can arise in other ways outside BER, for example, via a direct action of reactive oxygen species on DNA. SSBs coming into being in such cases also can be repaired through the BER pathway. Additionally, we tested the activity of Polβ on substrate 19/36, which does not mimic any BER intermediate but is a common model DNA substrate in Polβ research [25,26,27].

**Figure 2:**
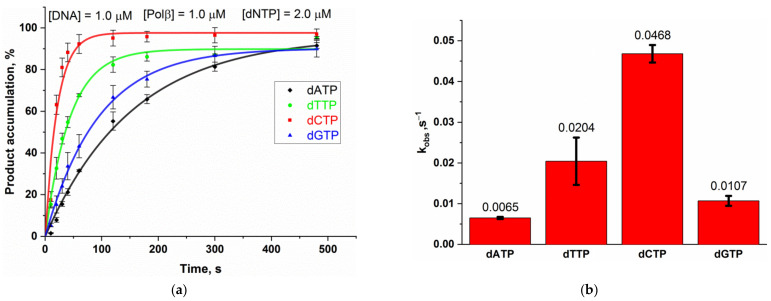
The assay of Polβ’s preference for dNTPs during dNTP incorporation. (**a**) The kinetic time courses of Polβ-catalyzed single-nucleotide incorporation into four different 14/28 DNA substrates containing a different templating nucleotide that is complementary to A, T, C, or G. The experimental data points were fitted to Equation (2) to obtain observed rate constant *k*_obs_ for each dNTP. The original PAGEs are provided in the Supplementary Figure S1. (**b**) *k*_obs_ for incorporation of various nucleotides indicating a bias of Polβ in nucleotide incorporation.

**Figure 3:**
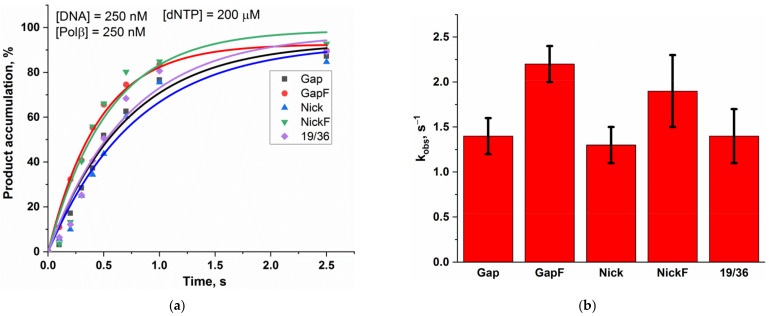
The assay of Polβ’s specificity to different DNA substrates. (**a**) Kinetic time courses of Polβ-catalyzed single-nucleotide incorporation into various DNA substrates. The experimental data points were fitted to Equation (2) to determine observed rate constant *k*_obs_ for each DNA substrate. The original PAGEs are provided in the Supplementary Figure S2. (**b**) *k*_obs_ for Polβ-catalyzed single-nucleotide incorporation into the different DNA substrates.

Before testing the activity of Polβ on the different DNA substrates, we investigated whether Polβ has any preference for a dNTP regarding its incorporation. For this purpose, we tested Polβ polymerase activity at low dNTP concentration (2 µM) on four substrates 14/28 (Figure 1) having the same structure but different template nucleotides. Polβ was found to differ in the efficiency of incorporation of dATP, dGTP, dCTP, and dTTP under these experimental conditions (Figure 2, Supplementary Figure S1). The incorporation efficiency of pyrimidines was higher than that of purines. Because of this difference, we performed experiments to compare Polβ polymerase activity toward different DNA substrates (Figure 3, Supplementary Figure S2) at saturating dNTP concentrations (200 µM) in order to minimize the effect of the dNTP nature on the rate of Polβ action.

Kinetic time courses of incorporation of a single nucleotide into the different model DNA substrates by Polβ are presented in Figure 3A. The rate constants of nucleotide incorporation into these substrates calculated by means of Equation (2) are given in Figure 3B. It was demonstrated that the rate of Polβ-catalyzed nucleotide incorporation into GapF is better than the rates toward Gap, Nick, and 19/36, consistent with the fact that GapF models the canonical substrate of Polβ in BER. For instance, Polβ polymerase activity is sensitive to the 5′-dRP group even if it could not form the Schiff base with it. It is worth mentioning that the rate of the nucleotide incorporation into substrate NickF is close to that of the incorporation into GapF, suggesting that Polβ may participate in the long-patch subpathway of BER by performing strand displacement DNA synthesis.

**Figure 4:**
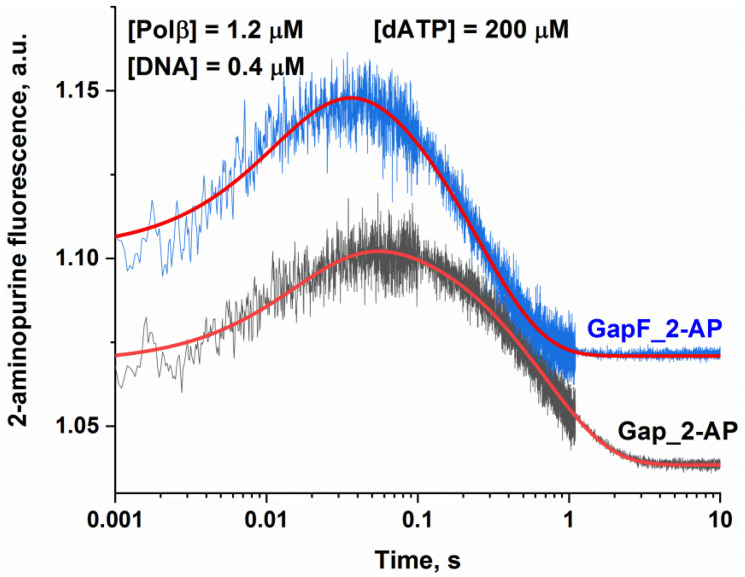
Changes in 2-aminopurine fluorescence intensity during the interaction of Polβ with DNA substrate Gap or GapF in the presence of dATP. Experimental data and results of two-exponential fitting (Equation (3)) are presented as jagged and smooth lines, respectively.

From previous structural [28,29] and kinetic [25,27,30] studies, it is known that Polβ undergoes global conformational rearrangements during its catalysis. To monitor these conformational dynamics, we used the DNA substrates containing a 2-aminopurine (2-AP) one base after the templating base; these substrates have been described previously as a good probe for the Polβ subdomain closing and reopening during its interaction with DNA [25,27,30]. The stopped-flow fluorescence trace of the interaction of Polβ (1.2 µM) with DNA substrates Gap_2-AP and GapF_2-AP (0.4 µM) is presented in Figure 4. In other articles [25,27,30], the changes in 2-aminopurine fluorescence in such stopped-flow fluorescence curves have been attributed to conformational alterations of the Polβ enzyme–substrate complex. The initial rapid fluorescence increase has been found to match the subdomain’s open-to-closed transition induced by nucleotide (Mg·dNTP^2−^) binding. The subsequent decrease in fluorescence corresponds to subdomain reopening after nucleotidyl transfer (i.e., chemical step), and the rate of this step has been shown to be limited by the chemical process under these conditions. A two-exponential fitting (Equation (3)) of these curves revealed meanings of the rate constants for DNA-binding and chemical steps, with chemical rate constants being close to those derived from the quenched-flow experiment with the respective substrates (Table 2). These results confirmed that the rate of the fluorescence decrease phase matches the rate of the chemical step. Stopped-flow fluorescence data also indicated that both the binding step and chemical step are faster for substrate GapF than for substrate Gap. This finding confirmed Polβ’s sensitivity to the 5′-dRP group even without Schiff base formation.

### 2.2. The Impact of DNA Glycosylases on Polβ Polymerase Activity as Revealed by Polyacrylamide Gel Electrophoresis (PAGE)

**Figure 5:**
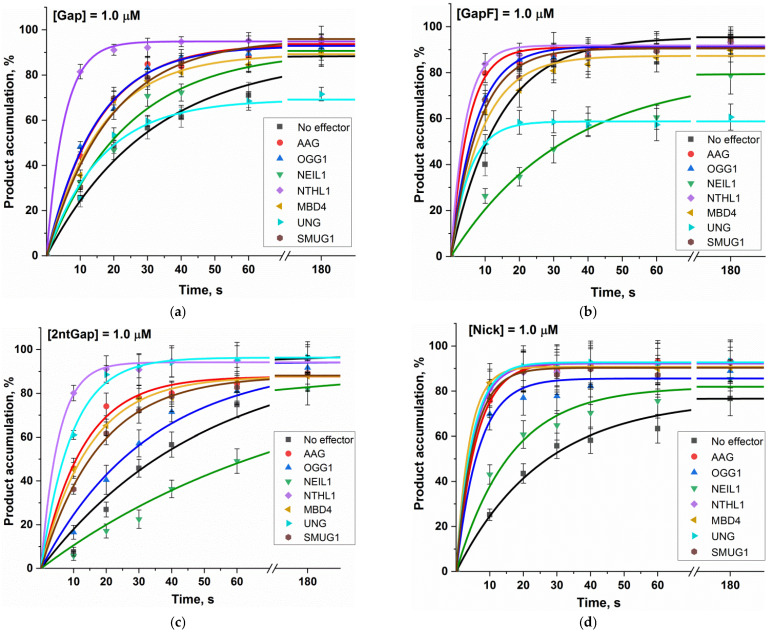
The kinetic time courses of Polβ-catalyzed single-nucleotide incorporation into DNA substrates (**a**) Gap, (**b**) GapF, (**c**) 2ntGap, and (**d**) Nick in the presence or absence of a DNA glycosylase. The concentrations of the DNA substrate and Polβ in a reaction mixture were 1.0 µM, and each DNA glycosylase was added to a final concentration of 2.0 µM. The experimental data points were fitted to Equation (2) to determine observed rate constant *k*_obs_ for the kinetic curve. The original PAGEs are provided in the Supplementary Figures S3–S6.

**Figure 6:**
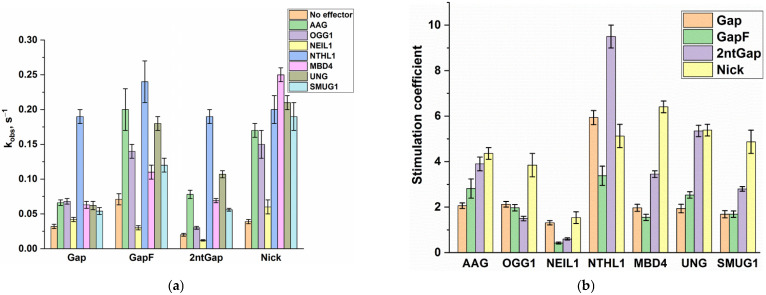
The influence of the different DNA glycosylases on the Polβ activity toward different DNA substrates. (**a**) The observed rate constants of Polβ-catalyzed single-nucleotide incorporation into different substrates in the absence or presence of a DNA glycosylase. (**b**) Stimulation coefficients for effects of DNA glycosylases on Polβ activity toward different DNA substrates, as computed according to Equation (1).

To understand how DNA glycosylases can interfere with the Polβ activity during BER, we implemented Polβ-catalyzed single-nucleotide incorporation into four different model DNA substrates (Gap, GapF, 2ntGap, and Nick) in the presence of one of seven human DNA glycosylases: UNG, SMUG1, MBD4, OGG1, NTHL1, AAG (also known as MPG), and NEIL1. The polymerase activity was evaluated by direct PAGE analysis of DNA product accumulation. Kinetic time courses of product accumulation are shown in Figure 5 (Supplementary Figures S3–S6). The exponential fitting of each curve yielded an observed rate constant (*k*_obs_). All these constants are presented in Table 3 and Figure 6A. A ratio of an observed constant in the presence of an effector protein to the observed rate constant in the absence of the effector protein for each substrate gave a stimulation coefficient, which characterizes an impact of each glycosylase on Polβ’s activity toward each DNA substrate:

(1) $$f_{E,S}=\frac{k_{obs}^{E,S}}{k_{obs}^{0,S}}$$

where $f_{E,S}$ is the stimulation coefficient for DNA glycosylase E and DNA substrate S, $k_{obs}^{E,S}$ is an observed rate constant of Polβ-catalyzed single-nucleotide incorporation into DNA substrate S in the presence of DNA glycosylase E, and $k_{obs}^{0,S}$ is the observed rate constant of Polβ-catalyzed single-nucleotide incorporation into DNA substrate S in the absence of any effector protein.

These stimulation coefficients for each DNA glycosylase and each substrate are presented in Figure 6B.

Overall, all the evaluated DNA glycosylases had a stimulatory effect on the Polβ polymerase activity toward all the four tested substrates. An exception is NEIL1, which did not have a significant effect in cases of substrates Gap and Nick and inhibited the activity of Polβ toward GapF and 2ntGap.

The greatest effect was observed in the case of NTHL1, which manifested a stimulation coefficient of 9.5 for substrate 2ntGap. Stimulation coefficients of this enzyme for the other DNA substrates proved to be in the range 3–6. It seemed that in the case of MBD4, UNG, and SMUG, stimulation coefficients for 2ntGap and Nick (which are in the range of 3–6) are significantly greater than those for Gap and GapF (which are ~2). In the meantime, in the case of AAG, stimulation coefficients for 2ntGap and Nick are only slightly greater than those for Gap and GapF. OGG1 was found to have a stimulation coefficient for Nick approximately twofold greater (~4) than stimulation coefficients for Gap, GapF, and 2ntGap (~2). Furthermore, it is worth noting that, although UNG enhanced the rate of Polβ-catalyzed single-nucleotide incorporation into Gap and GapF, it diminished the maximum level of substrate conversion to 60–70%.

Interactions between Polβ and DNA glycosylases during BER could be thought of in terms of both the “passing the baton” model and preassembled repair complexes. In contrast to the “classic” case of BER, where damaged DNA is directly passed on from a monofunctional DNA glycosylase to APE1, it is possible that an SSB formed by a bifunctional DNA glycosylase is directly passed on to Polβ. Indeed, Polβ is reported to specifically displace OGG1 and NEIL1 from damaged DNA [20], thereby supporting this hypothesis. Nonetheless, there is a problem with this explanation: the damaged DNA processed by a bifunctional DNA glycosylase has a 3′-blocking group, which does not allow Polβ to incorporate a nucleotide. This 3′ end can be processed by APE1 [31] or polynucleotide kinase phosphatase (PNKP) (in the case of 3′-phosphate) [32]. Accordingly, we can propose that Polβ and/or a bifunctional DNA glycosylase can engage in direct protein–protein interactions to recruit a 3′-end processor to a damaged DNA or to form a preassembled multiprotein complex that can efficiently repair the damaged DNA via coordination between BER participants. Such multiprotein repair complexes may involve a bifunctional or monofunctional DNA glycosylase. Indeed, immunoprecipitation analyses suggest that monofunctional DNA glycosylases, such as UNG [18] or AAG [19], or bifunctional ones, such as OGG1 [19], NEIL1 [22], or NEIL2 [23], are present in multiprotein repair complexes with Polβ. What is more, NEIL2 and Polβ are believed to be associated in the multiprotein complex with PNKP, which can process a 3′-phosphate blocking group generated by NEIL2 [23]. Besides, UNG and Polβ are associated in the multiprotein complex with APE1, which can process an AP site after UNG to prepare the damaged DNA for Polβ [18]. It has been shown that NEIL2′s domain involved in interactions with Polβ and other proteins is required for efficient DNA repair [23]. Hence, it can be proposed that DNA glycosylases and Polβ can affect each other’s enzymatic activities during BER.

In the current study, we demonstrated that human DNA glycosylases AAG, OGG1, NTHL1, MBD4, UNG, and SMUG1 enhance the nucleotidyl transferase activity of Polβ toward different model DNA substrates imitating intermediates that can arise during BER. Because we carried out the Polβ reactions under single-turnover conditions, this finding cannot be explained by possible displacement of Polβ from its product by DNA glycosylases. Furthermore, because the DNA substrates that we used are not specific for any DNA glycosylase, it is unlikely that DNA glycosylases can recruit Polβ to these DNA lesions. Consequently, we propose that Polβ forms protein–protein contacts with DNA glycosylases to enhance its nucleotidyl transferase activity. Possibly, these interactions are formed in multiprotein complexes containing Polβ and DNA glycosylase. This idea is consistent with previously published evidence of such complexes for UNG [18], AAG [19], and OGG1 [19]. Polβ probably forms such complexes both before and after DNA binding. In this way, our results support the notion that interactions between Polβ and DNA glycosylases involve multiprotein repair complexes for enhancement of the efficiency of DNA repair.

Some of our experimental findings differ from commonly reported results and should be discussed in detail. For example, we observed a slowing of the rate of Polβ’s activity toward GapF and 2ntGap by NEIL1 and diminished levels of Gap and GapF substrate conversion when UNG was added. In terms of assembly of protein–protein complexes, these findings can be explained as follows: a complex between Polβ and NEIL1 or UNG can have lower affinity for the corresponding substrates as compared to free Polβ.

It is also noteworthy that nucleotide incorporation into substrate Nick matches the long-patch subpathway of BER [5]. Therefore, the stimulation of this reaction by all the tested DNA glycosylases indicates that DNA glycosylases may switch the direction of the BER pathway, thereby making Polβ perform strand displacement synthesis. Replicative Polδ is believed to continue strand displacement synthesis typically during the long-patch subpathway of BER [5,13], whereas Polβ is thought to only incorporate the first nucleotide. On the other hand, an ability of Polβ to perform strand displacement synthesis in vitro has been documented previously [14] and in the current study.

## 3. Materials and Methods

### 3.1. Protein Expression and Purification

Human DNA polymerase β (Polβ) was expressed in Rosetta 2 (DE3) *Escherichia coli* cells. The cells carrying a pET28c expression vector were grown at 37 °C in 1 L of the Luria–Bertani (LB) medium supplemented with 50 μg/mL kanamycin to an optical density of 0.6 at 600 nm (A_600_). Then, the temperature was lowered to 20 °C, and transcription was induced by the addition of 0.2 mM isopropyl β-d-1-thiogalactopyranoside. After that, the cells were incubated for 16 h. The cells were harvested by centrifugation (5000× *g*, 10 min) and then resuspended in a buffer (20 mM HEPES-KOH pH 7.8, 40 mM NaCl) followed by cell lysis by means of a French press. All the purification procedures were carried out at 4 °C. Each homogenate was centrifuged at 40,000× *g* for 40 min, and the supernatant was passed through a column packed with 30 mL of the Q-Sepharose resin (Amersham Biosciences) and pre-equilibrated in a buffer (20 mM HEPES-KOH pH 7.8, 200 mM NaCl). The flow-through fractions containing the Polβ protein were pooled, supplemented with 20 mM imidazole, and loaded onto a 1 mL HiTrap-Chelating™ column (GE HealthCare, Chicago, IL, USA). Bound proteins were eluted with a linear 20 → 500 mM gradient of imidazole.

NTHL1 was expressed in Rosetta 2 (DE3) *E. coli* cells carrying a pET14b expression vector. The cells were grown at 37 °C in 1 L of the LB medium supplemented with 100 μg/mL ampicillin to A_600_ of 0.6. Then, the expression and purification of NTHL1 were the same as described above for Polβ.

Human DNA glycosylases UNG, SMUG1, MBD4 (catalytic-domain–containing residues 426–580), OGG1, AAG, and NEIL1 were expressed and purified as described previously [33,34,35,36,37,38].

The proteins’ concentrations were measured by means of A_280_; their stock solutions were stored at −20 °C in 50% glycerol.

### 3.2. Oligodeoxynucleotides

Sequences of the DNA substrates employed in this work are presented in Figure 1. The substrates were prepared by annealing a complementary chain (or the respective upstream and downstream primers) to a template oligonucleotide mixed in the equimolar ratio. Oligodeoxynucleotides were synthesized by standard phosphoramidite methods on an ASM-800 synthesizer (BIOSSET Ltd., Novosibirsk, Russia) using phosphoramidites purchased from either Glen Research or ChemGenes. The synthetic oligodeoxynucleotides were purified by high-performance liquid chromatography on an Agilent 1200 chromatograph (USA) and a Zorbax SB-C18 column (5 μm), 4.6 mm × 150 mm, via a linear gradient of acetonitrile (0 → 50%) in the presence of 20 mM triethylammonium acetate (pH 7.0) for 30 min at a flow rate of 2 mL/min. Fractions containing oligodeoxynucleotides were dried in vacuum, dissolved in water, and precipitated with 2% LiClO_4_ in acetone. After a wash with pure acetone and drying, the oligodeoxynucleotide precipitates were dissolved in water and stored at −20 °C until the experiments. Concentrations of the oligodeoxynucleotides were determined through the use of A_260_. Homogeneity of the purified oligodeoxynucleotides was evaluated by PAGE in a denaturing 20% gel. The oligodeoxynucleotides were visualized with the Stains-All dye (Sigma-Aldrich, Burlington, MA, USA).

### 3.3. Polβ Polymerase Activity Assays Using PAGE Analysis

To study (a) Polβ’s preference for dNTPs during dNTP incorporation, (b) the specificity of Polβ to different DNA substrates, and (c) the impact of DNA glycosylases on Polβ activity, we assayed Polβ-catalyzed single-nucleotide incorporation into DNA using chemical quench experiments with PAGE analysis. To start a reaction, a solution of Polβ was mixed with a solution of a DNA substrate in a reaction buffer consisting of 50 mM Tris-HCl pH 7.5, 50 mM KCl, 1.0 mM EDTA, 5.0 mM MgCl_2_, 1.0 mM DTT, 7% of glycerol, and typically 2 µM deoxynucleotide triphosphate (dNTP) corresponding a template nucleotide in a DNA substrate. An exception was the analysis of specificity of Polβ to different DNA substrates (Figure 3), where we used dNTP’s concentrations of 200 µM. Concentrations of Polβ and a DNA substrate in the reaction mixture were 1.0 µM, except for the analysis of specificity of Polβ to various DNA substrates (Figure 3), where concentrations of the enzyme and a DNA substrate were 250 nM. When a DNA glycosylase was added, its concentration in the reaction mixture was 2.0 µM.

The reactions were carried out either at 25 °C (in the assays of Polβ’s preferences in dNTP incorporation and specificity to different DNA substrates) or at 37 °C (in the analyses of the impact of DNA glycosylases on Polβ activity). At certain time points, the reactions were quenched with a stop solution composed of 8 M urea and 20 mM EDTA and then loaded on a 20% (*w*/*v*) polyacrylamide gel containing 7 M urea. The gels were visualized via fluorescence of FAM at the end of an oligonucleotide primer extended by Polβ using an E-Box CX.5 TS gel-documenting system (Vilber Lourman, France) and were quantified in the Gel-Pro Analyzer 4.0 software (Media Cybernetics, Rockville, MD, USA).

When Polβ’s specificity to various DNA substrates (Figure 3) was investigated at 200 µM dNTP, the reactions were allowed to proceed for periods ranging from 100 ms to 2.5 s with the help of a rapid chemical quench instrument (KinTek Corp., State College, PA, USA).

Kinetic time courses of accumulation of single-nucleotide incorporation products were fitted to a single-exponential curve using the OriginPro 2018 software (Originlab Corp., Northampton, MA, USA):

(2) $$[\mathrm{product}]=A\;\text{×}\;[1\;-\;\exp(-k_{obs}t)]$$

where A is the amplitude, *k*_obs_ is an observed rate constant, and t is the reaction time.

### 3.4. The Stopped-Flow Assay

Stopped-flow measurements with fluorescence detection were performed mostly as described elsewhere [34,35,39] by means of a model SX.20 stopped-flow spectrometer (Applied Photophysics Ltd., Leatherhead, UK). The fluorescence of 2-aminopurine was excited at λ_ex_ = 310 nm and monitored at λ_em_ > 370 nm as transmitted by the LG-320 filter (Corion, Franklin, MA, USA). Typically, each trace shown is the average of four or more individual experiments.

A reaction was started by mixing a solution of the enzyme with a solution of a DNA substrate (Gap_2-AP or GapF_2-AP) to final concentrations 1.2 and 0.4 µM for the enzyme and DNA, respectively. The reactions were conducted at 37 °C in reaction buffer consisting of 200 µM dATP, 50 mM Tris-HCl pH 7.5, 50 mM KCl, 1.0 mM EDTA, 5.0 mM MgCl_2_, 1.0 mM DTT, and 7% of glycerol.

The stopped-flow curves were fitted to the double-exponential equation:

(3) $$F=F_{0}+F_{1}\left(1-\exp\left(-k_{1}t\right)\right)+F_{2}\left(1-\exp\left(-k_{2}t\right)\right),$$

where F is the observed fluorescence, $F_{0}$ is the background fluorescence, $F_{1}$ and $F_{2}$ are the fluorescence parameters, and $k_{1}$ and $k_{2}$ are observed rate constants of the first stage and second stage, respectively.

## 4. Conclusions

In this work, we assayed Polβ’s nucleotidyl transferase activity toward different DNA substrates that imitate DNA intermediates emerging during BER. It was demonstrated that at low dNTP concentrations, Polβ incorporates pyrimidines into DNA faster than purines. To assay Polβ’s activity at high dNTP concentrations, we took advantage of rapid-quench-flow and stopped-flow fluorescence approaches. Polβ was found to effectively incorporate a single nucleotide into different types of SSBs both with and without a 5′-dRP–mimicking group. It is worth noting that the nucleotide incorporation into one of these substrates corresponds to possible strand displacement synthesis by Polβ in the long-patch subpathway of BER. Consequently, the participation of Polβ in the long-patch subpathway in the BER mechanism cannot be ruled out. Moreover, we showed that Polβ incorporates a nucleotide more efficiently into SSBs containing a 5′-dRP–mimicking group even if this group cannot form a Schiff base with the enzyme.

In an attempt to fill gaps in the understanding of BER coordination, we investigated the effects of DNA glycosylases on Polβ’s nucleotidyl transferase activity toward different types of SSBs. Generally, various DNA glycosylases (AAG, OGG1, NTHL1, MBD4, UNG, and SMUG1) were found to stimulate Polβ’s activity on different DNA substrates. We explain these experimental findings by the concept of preformed stable multiprotein repair complexes (“repairosomes”) [15] rather than by the “passing the baton” model, which involves the emergence of transient protein complexes. Our explanation is consistent with previous assays revealing direct protein–protein interactions between Polβ and some DNA glycosylases and pointing to the existence of multiprotein complexes containing both Polβ and some DNA glycosylases [18,19,22,23]. Overall, it could be concluded that the formation of either transient or stable multiprotein complexes in the course of BER significantly affects individual enzymatic activities, whereas the structural arrangement and the mechanism of molecular functioning of such complexes are hot areas in the field of DNA repair research at present.

## Figures and Tables

**Table 1:** Human DNA glycosylases.

Enzyme	Substrate Specificity	Structural Superfamily	Mono- (M) or Bi- (B) Functional *	Known Interaction with Polβ
UNG	U in single-stranded (ss) and double-stranded (ds) DNA	α/β-fold	M	Interaction has been revealed by an immunoprecipitation assay [18].
SMUG1	U in ss- and dsDNA	α/β-fold	M	–
TDG	T, U, 3,N^4^-ethenoC, and oxidized/deaminated derivatives of 5-methylC opposite to G in dsDNA	α/β-fold	M	–
MBD4	T and U opposite to G in dsDNA	HhH	M	–
NTHL1	Oxidized pyrimidines in dsDNA	HhH	B	–
MYH	A and 2-OH-A opposite to G or 8-oxoguanine in dsDNA	HhH	M	–
OGG1	8-oxoguanine and FapyG opposite to C in dsDNA	HhH	B	Interaction has been detected by an immunoprecipitation assay [19]. It has been shown that Polβ can displace OGG1 from DNA [20].
AAG	Ring-alkylated purines, hypoxanthine, and 1,N6-ethenoA in ss and dsDNA	FMT_C	M	Interaction has been registered by an immunoprecipitation assay [19].
NEIL1	Oxidized pyrimidines and purines, ring-open N7-alkylated G modifications, and psoralen cross-links in ss- and dsDNA	H2TH	B	Interaction has been revealed by far-western [21] and immunoprecipitation analyses [22]. Amino acid residues 312–349 of NEIL1 and an N-terminal part of Polβ (residues 1–140) are reported to be critical for this interaction [22]. It has been found that Polβ can displace NEIL1 from DNA [20].
NEIL2	Oxidized pyrimidines and purines in bubble DNA	H2TH	B	Interaction has been revealed by far-western and immunoprecipitation analyses. It has been shown that the N-terminal domain of NEIL2 (amino acid residues 1–198) interacts with an N-terminal part of Polβ (residues 1–140) [23].
NEIL3	Oxidized pyrimidines and purines in ssDNA	H2TH	B	–

* DNA glycosylases that possess only N-glycosyl hydrolase activity are called monofunctional. DNA glycosylases having also intrinsic β-lyase activity are termed bifunctional. In addition to removing a damaged DNA base, such bifunctional DNA glycosylases can cleave the phosphodiester backbone 3′ to the AP site to generate a nick with 3′α,β-unsaturated aldehyde [5,24].

**Table 2:** Kinetic rate constants (mean ± SD) for the interaction between Polβ and DNA substrates, as derived from 2-aminopurine stopped-flow fluorescence (SFF) traces. Constant *k*_1_ corresponds to the step of the binding between Polβ and DNA, and *k*_2_ characterizes the chemical step of the nucleotidyl transferase reaction. For comparison, Gap’s and GapF’s *k*_obs_ values derived from the rapid quench flow (RQF) experiments with PAGE analysis are presented too.

	*k*_1_ (SFF), s^−1^	*k*_2_ (SFF), s^−1^	*k*_obs_ (PAGE-RQF), s^−1^
Gap_2-AP	58 ± 1	1.44 ± 0.01	1.4 ± 0.2
GapF_2-AP	73 ± 1	3.96 ± 0.02	2.2 ± 0.2

**Table 3:** Observed rate constants *k*_obs_ (s^−1^) of Polβ-catalyzed single-nucleotide incorporation into different DNA substrates in the absence or presence of various DNA glycosylases.

	No Effector	AAG	OGG1	NEIL1	NTHL1	MBD4	UNG	SMUG1
Gap	0.032 ± 0.003	0.066 ± 0.004	0.068 ± 0.004	0.042 ± 0.003	0.19 ± 0.01	0.063 ± 0.005	0.062 ± 0.006	0.054 ± 0.005
GapF	0.071 ± 0.008	0.20 ± 0.03	0.14 ± 0.01	0.030 ± 0.003	0.24 ± 0.03	0.11 ± 0.01	0.18 ± 0.01	0.12 ± 0.01
2ntGap	0.020 ± 0.002	0.078 ± 0.006	0.030 ± 0.002	0.012 ± 0.001	0.19 ± 0.01	0.069 ± 0.003	0.107 ± 0.005	0.056 ± 0.002
Nick	0.039 ± 0.003	0.17 ± 0.01	0.15 ± 0.02	0.06 ± 0.01	0.20 ± 0.02	0.25 ± 0.01	0.21 ± 0.01	0.19 ± 0.02

## Data Availability

Raw experimental data are available from N.A.A.K. upon request. Tel. +7 (383) 363-5175, E-mail: nikita.kuznetsov@niboch.nsc.ru.

## Supplementary Materials

The following supporting information can be downloaded at: https://www.mdpi.com/article/10.3390/ijms24119594/s1.
