# Supplementary material for: The Impact of Human DNA Glycosylases on the Activity of DNA Polymerase β toward Various Base Excision Repair Intermediates
Source: Int J Mol Sci. 2023 May 31;24(11):9594. doi: 10.3390/ijms24119594 (PMC10253626; doi:10.3390/ijms24119594)
Supplement: Supplementary file 1 [file ijms-24-09594-s001.zip › ijms-2389287-supplementary.pdf]

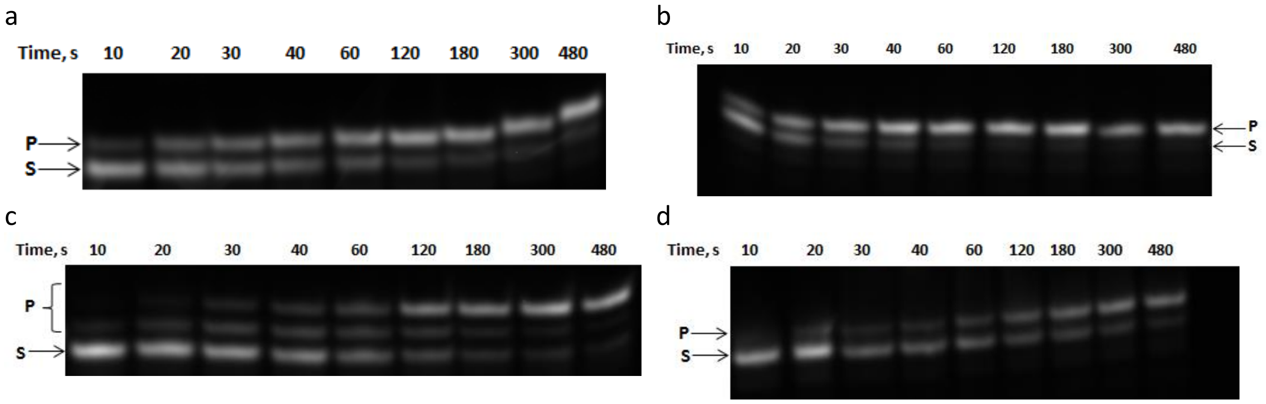

Figure S1. The PAGE kinetic time courses of Polβ-catalyzed single-nucleotide incorporation into four different 14/28 DNA substrates containing (a) A, (b) G, (c) C or (d) T as a templating nucleotide. S is a substrate, P is a product of single nucleotide incorporation. In the case of G on the PAGE there are both the product of single nucleotide incorporation and the product of incorporation of two nucleotides. Because of the second nucleotide can be incorporated only into the product of the single nucleotide incorporation, a total amount of single nucleotide incorporation product was counted as a sum of both products amounts.

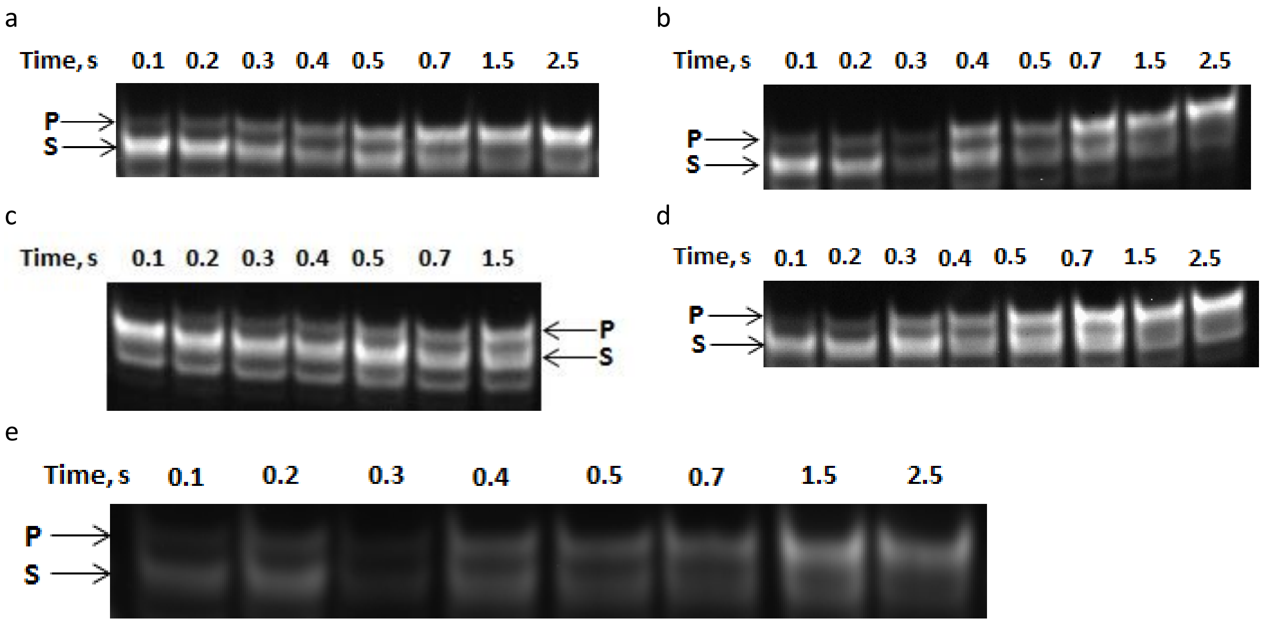

Figure S2. The PAGE kinetic time courses of Polβ-catalyzed single-nucleotide incorporation into (a) Gap, (b) GapF, (c) Nick, (d) NickF, (e) 19/36 DNA substrates. S is a substrate, P is a product of single nucleotide incorporation.

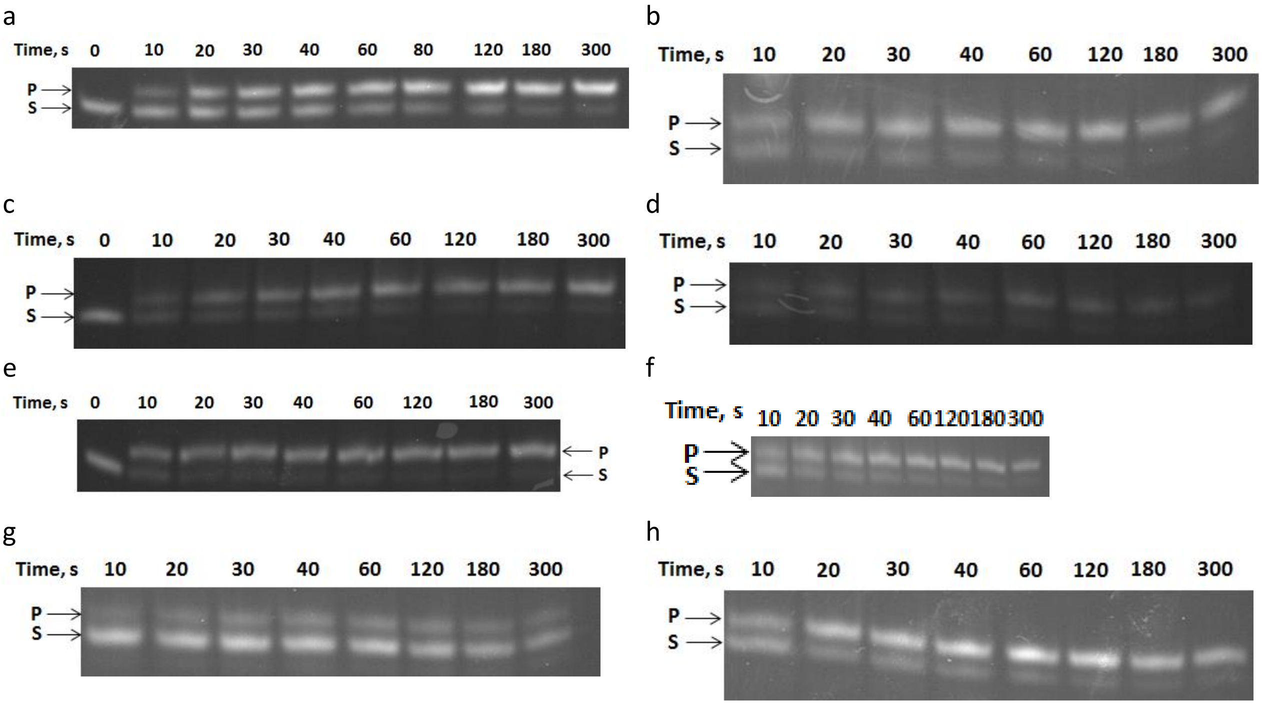

Figure S3. The PAGE kinetic time courses of Polβ-catalyzed single-nucleotide incorporation into Gap (a) in absence of any effectors or in presence of (b) AAG, (c) OGG1, (d) NEIL1, (e) NTHL1, (f) MBD4, (g) UNG, (h) SMUG1. S is a substrate, P is a product of single nucleotide incorporation.

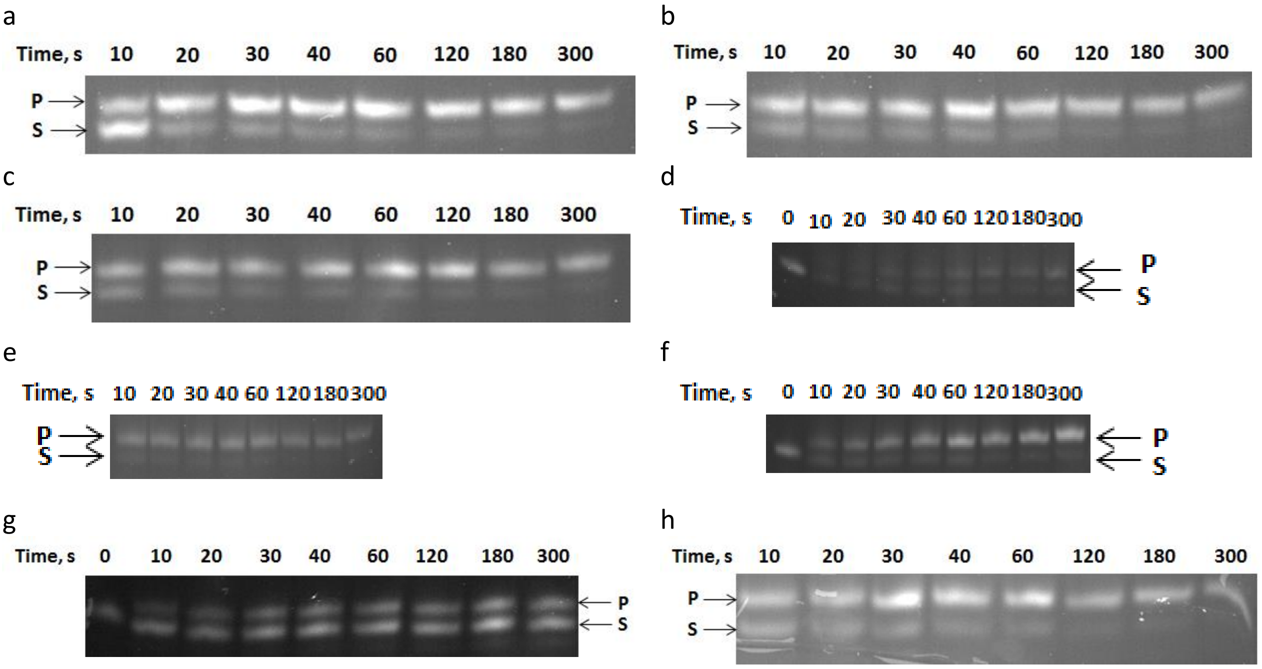

Figure S4. The PAGE kinetic time courses of Polβ-catalyzed single-nucleotide incorporation into GapF (a) in absence of any effectors or in presence of (b) AAG, (c) OGG1, (d) NEIL1, (e) NTHL1, (f) MBD4, (g) UNG, (h) SMUG1. S is a substrate, P is a product of single nucleotide incorporation.

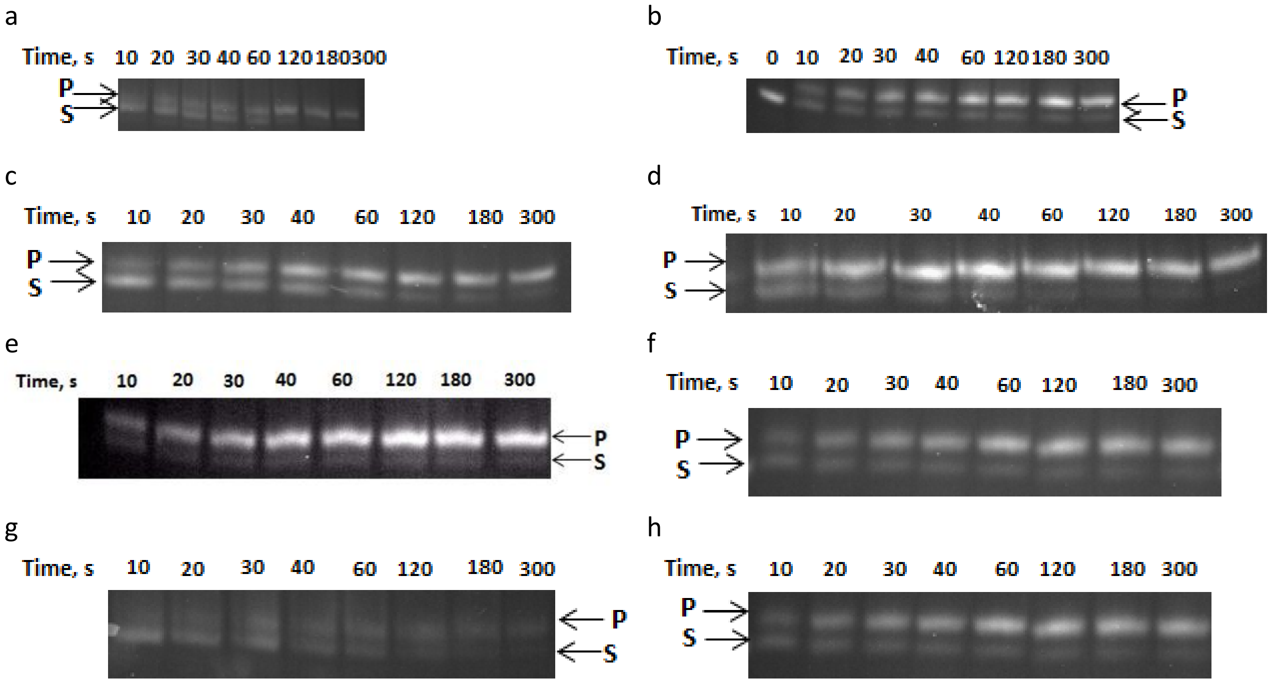

Figure S5. The PAGE kinetic time courses of Polβ-catalyzed single-nucleotide incorporation into 2ntGap (a) in absence of any effectors or in presence of (b) AAG, (c) OGG1, (d) NEIL1, (e) NTHL1, (f) MBD4, (g) UNG, (h) SMUG1. S is a substrate, P is a product of single nucleotide incorporation.

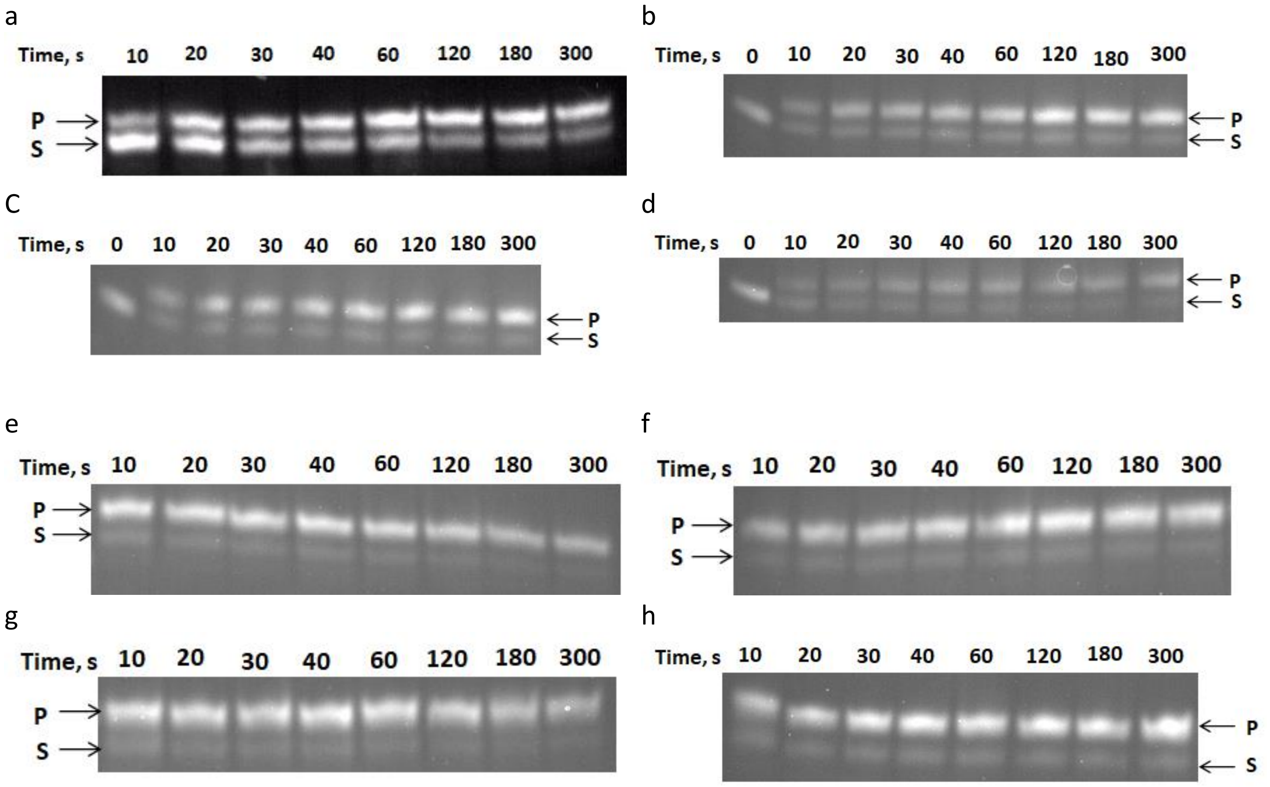

Figure S6. The PAGE kinetic time courses of Polβ-catalyzed single-nucleotide incorporation into Nick (a) in absence of any effectors or in presence of (b) AAG, (c) OGG1, (d) NEIL1, (e) NTHL1, (f) MBD4, (g) UNG, (h) SMUG1. S is a substrate, P is a product of single nucleotide incorporation.
